# Imatinib treatment improves hyperglycaemic dysregulation in severe COVID-19: a secondary analysis of blood biomarkers in a randomised controlled trial

**DOI:** 10.1186/s13054-024-04829-y

**Published:** 2024-02-29

**Authors:** Erik Duijvelaar, Xiaoke Pan, Harm Jan Bogaard, Etto C. Eringa, Jurjan Aman

**Affiliations:** 1 Amsterdam University Medical Centers, location Vrije Universiteit Amsterdam, Department of Pulmonary Medicine, Amsterdam Cardiovascular Sciences, Amsterdam, The Netherlands; 2 Department of Physiology, Amsterdam Cardiovascular Sciences, Amsterdam University Medical Centers, Amsterdam, The Netherlands; 3 Department of Physiology, Maastricht University, Cardiovascular Research Institute Maastricht, Maastricht, The Netherlands

**Keywords:** COVID-19, Insulin resistance, Respiratory failure, Proteomics

## Abstract

SARS-CoV-2 can induce insulin resistance, which is, among others, mediated by adipose tissue dysfunction and reduced angiotensin-converting enzyme 2 (ACE2) enzymatic activity. In SARS-CoV-2-infected mice, the tyrosine kinase inhibitor imatinib attenuates inflammation and improves insulin sensitivity. Here, we report the effects of imatinib on incident hyperglycaemia, circulating levels of glucoregulatory proteins, longitudinal insulin sensitivity and ACE-2 enzymatic activity in 385 hospitalized COVID-19 patients who participated in a randomized, double-blind, placebo-controlled clinical trial. Patients with severe hyperglycaemia had similar demographics compared to those without, but required longer hospital stays and exhibited higher invasive ventilation and mortality rates. The incidence of severe hyperglycaemia was significantly lower in patients treated with imatinib, while insulin production and central insulin sensitivity were unaffected. Imatinib increased plasma angiotensin-2 and adiponectin levels, and decreased c-Jun N-terminal protein kinase 1 (JNK1), JNK2 and interleukin-6 levels. These findings suggest that imatinib restores endocrine control of peripheral glucose uptake in COVID-19.

**Supplementary Information:**

The online version contains supplementary material available at 10.1186/s13054-024-04829-y.

## Main text

Severe acute respiratory syndrome coronavirus 2 (SARS-CoV-2) infections can induce insulin resistance, which is often exacerbated by glucocorticoid treatment [1]. SARS-CoV-2-induced insulin resistance is among others mediated by adipose tissue dysfunction and suppression of angiotensin-converting enzyme 2 (ACE2) enzymatic activity [1, 2]. In a study on SARS-CoV-2-infected mice, the tyrosine kinase inhibitor imatinib was found to attenuate inflammation and improve insulin sensitivity [2]. Here, we present the effects of imatinib treatment on glucose metabolism in patients with severe COVID-19.

This secondary study utilized clinical and blood sample data from a randomized, double-blind, placebo-controlled clinical trial that assessed the efficacy of oral imatinib treatment in hospitalized hypoxemic COVID-19 patients [3, 4]. While the trial did not meet its primary endpoint of time to liberation from ventilation and oxygen supplementation oxygen, imatinib-treated patients exhibited a shorter duration of invasive ventilation and lower mortality rate [3]. Furthermore, the incidence of grade 3 or higher hyperglycaemia, defined as the initiation of insulin therapy according to the Common Terminology Criteria for Adverse Events (CTCAE) version 5.0, was lower in imatinib-treated patients (11% vs. 20%, Chi-squared test *p* = 0.022). The mechanisms underlying these beneficial effects are unknown. Therefore, we investigated the effects of imatinib on circulating glucoregulatory proteins, longitudinal insulin sensitivity and ACE-2 enzymatic activity.

During the trial, glucose levels were measured upon hospital admission and consequently once daily for all patients according to the in-hospitals protocols of the 13 participating hospitals. The frequency was increased to three to four times daily after the start of dexamethasone treatment, and in the case of preexisting diabetes mellitus or hyperglycaemic dysregulation. Insulin therapy was initiated when glucose levels exceeded 12 mmol/L (≈ 215 mg/dL) or were consistently above 10 mmol/L (≈ 180 mg/dL). Blood samples were systematically collected and stored at − 80 °C. Samples collected at hospital admission, before first investigational drug administration, were used for baseline assessments. Samples collected after three days, or two days if unavailable, of treatment were used for follow-up assessments. Patients were allowed to eat without restriction. Therefore, these samples were typically not collected under fasting conditions. Plasma levels of glucoregulatory proteins were quantified using modified aptamers (Somalogic Inc., USA). A panel of 34 glucoregulatory proteins was curated from the SomaScan library version 4.1, involving literature referencing and exploration of pertinent biological pathways through the Kyoto Encyclopedia of Genes and Genomes (KEGG) and Reactome databases [5, 6]. Serum glucose, insulin and C-peptide levels were measured using in-hospital laboratory enzyme-linked immunosorbent assays (ELISA) of the Amsterdam University Medical Center. ACE-2 enzymatic activity was measured in follow-up plasma samples with angiotensin (Ang)-II and Ang(1-7) ELISA kits (CUSABIO Biotech, China) in a random subgroup of 76 patients matched for sex, obesity, diabetes mellitus, need for invasive ventilation and mortality. Linear mixed models were employed to analyse protein abundance over time. These models incorporated hospital site, treatment, time and time × treatment interaction as fixed effects and random intercepts for each patient. Contrasts were applied for comparisons within treatment and time groups. The *p*-value of the time × treatment interaction (*p*_interaction_) was used to assess the effect of imatinib over time. The Benjamini–Hochberg method with a false discovery rate of 0.05 was used to correct *p*-values for multiple testing.

**Fig. 1:**
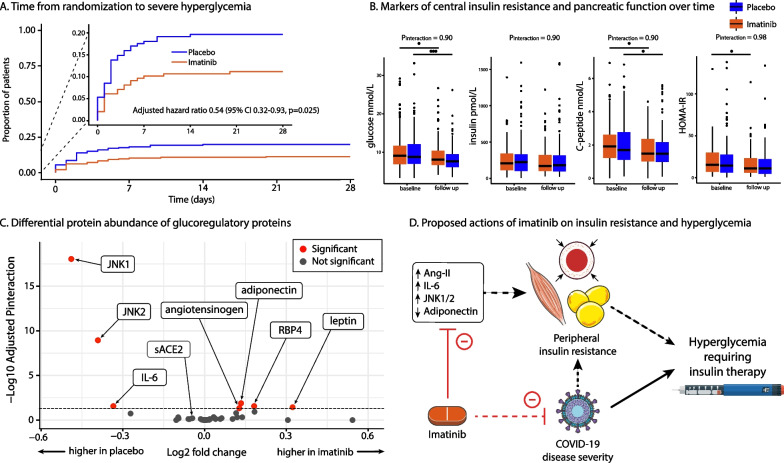
**A** Kaplan–Meier curve of time to severe hyperglycaemia development according to the Common Terminology Criteria for Adverse Events (CTCAE) version 5.0. The hazard ratio was calculated using Cox proportional hazards regression and was adjusted for comorbid diabetes mellitus. **B** Markers of central insulin resistance and pancreatic function over time. The centre line represents the median, the lower and upper bound represent the interquartile range. *P*_interaction_ values were derived from linear mixed models and were corrected for multiple testing. HOMA-IR = Homeostatic Model Assessment for Insulin Resistance. **C** Volcano plot of differential protein abundance using proteomics performed by Somalogic Inc., with modified aptamers. Each dot represents a single protein. *p*-values were adjusted using the Benjamini–Hochberg method. **D**. COVID-19 disease severity is associated with peripheral insulin resistance and hyperglycaemic dysregulation. Inflammation, JNK deficiency, adipose tissue dysfunction and impaired microvascular perfusion are associated with insulin resistance. Treatment with imatinib improves IL-6, Ang-II, JNK1, JNK2 and adiponectin levels, and increases microvascular perfusion. The solid lines indicate that the relationship can be inferred from the findings from our study. Dashed lines indicates that the association can only be inferred from external studies. Ang-II = angiotensin II, IL-6 = InterLeukin-6, JNK1 = c-Jun N-terminal protein kinase 1, JNK2 = c-Jun N-terminal protein kinase 2

In a Cox proportional hazards model adjusted for diabetes mellitus, imatinib treatment was associated with a reduced hazard of severe hyperglycaemia and subsequent need for insulin administration (hazard ratio (HR) 0.54, 95% CI 0.32–0.93; *p* = 0.025; Fig. 1A). Correcting for diabetes mellitus, cardiovascular disease, obesity and sex yielded similar results (HR 0.55, 95% CI 0.32–0.96; *p* = 0.036). Notably, patients with severe hyperglycaemia had similar demographics compared to those without, but required longer hospital stays and exhibited higher invasive ventilation and mortality rates (*p* < 0.001 for all; Table 1). Samples from 162 patients treated with imatinib and 156 patients treated with placebo were included for blood analysis. The mean age of participants at baseline was 64 years, mean BMI was 29.2 kg/m^2^, 30% were female and 28% had diabetes mellitus. At hospital admission, patients who eventually developed severe hyperglycaemia exhibited higher levels of interleukin-8, soluble receptor for advanced glycation end products (RAGE) and interleukin-6, and lower Jun dimerization protein 2 abundance (Table 1). Levels of glucose, insulin, C-peptide and the HOMA-IR were not different at hospital admission. C-peptide and glucose levels decreased over time in both treatment groups (Fig. 1B). HOMA-IR decreased significantly in patients treated with imatinib, while the change in patients treated with placebo was non-significant. Insulin levels remained stable in both groups. Imatinib treatment did not affect glucose, C-peptide, insulin or HOMA-IR levels (*p*_interaction_ are 0.80, 0.80, 0.80 and 0.92, respectively; Fig. 1B). Patients treated with imatinib had lower Ang-II levels (*p* = 0.038) at follow-up, but Ang(1-7) levels did not change (not shown in Figure). Ang-II/Ang(1-7) ratio, a surrogate marker of ACE2 enzymatic activity, was not different between the treatment groups (*p* = 0.17; not shown in Figure). At baseline, plasma levels of all glucoregulatory proteins were similar between the two groups. Imatinib treatment significantly decreased plasma levels of interleukin-6 (*p*_interaction_ = 0.026), c-Jun N-terminal protein kinase 1 (JNK1) and JNK2 (*p*_interaction_ < 0.0001 for both; Fig. 1C, Additional file 1: Table S1). Imatinib increased angiotensinogen (*p*_interaction_ = 0.044) and adiponectin (*p*_interaction_ = 0.012) levels (Additional file 1: Table S1). Patients treated with imatinib had a smaller reduction in leptin (*p*_interaction_ = 0.034) and a larger increase in retinol-binding protein 4 (*p*_interaction_ = 0.026) levels over time, but protein abundances were not different at follow-up (Additional file 1: Table S1). Imatinib did not affect soluble ACE2 levels (*p*_interaction_ = 0.44; Additional file 1: Table S1).

**Table 1:** Characteristics of patients who developed severe hyperglycaemia versus those who did not

Characteristic	No hyperglycemia *n* = 326	Hyperglycemia *n* = 59	*p*-value
Age—mean (SD)	63.8 (12.6)	65.7 (11.7)	0.28
BMI—mean (SD)	29.5 (5.5)	28.9 (5.2)	0.47
Males—no. (%)	222 (68)	42 (71)	0.75
*Comorbidities—no. (%)*			
Current/former smoker	121 (42)	23 (42)	1.00
Diabetes mellitus, any type	83 (26)	17 (29)	0.71
Chronic kidney disease	11 (3)	3 (5)	0.78
Hypertension	123 (38)	22 (37)	1.00
Dexamethasone treatment—no. (%)	225 (69)	51 (86)	0.01
*Clinical outcomes*			
Need for intensive care admission—no. (%)	43 (13)	29 (49)	< 0.001
Need for invasive ventilation—no. (%)	32 (10)	24 (41)	< 0.001
Days of hospital admission—median [IQR]	6 [3–9]	15 [8–23]	< 0.001
Mortality—no. (%)	33 (10)	16 (27)	< 0.001
*Markers of insulin resistance and beta cell function at hospital admission*			
insulin in pmol/L, median [IQR]	213.3 [105.2, 336.5]	224.6 [107.8, 349.1]	0.796
C-peptide in nmol/L, median [IQR]	1.8 [1.1, 2.6]	2.3 [1.2, 3.0]	0.144
glucose in mmol/L, median [IQR]	8.8 [6.8, 11.5]	9.4 [8.0, 13.1]	0.067
HOMA-IR (median [IQR])	14.8 [6.3, 29.6]	17.3 [8.2, 26.7]	0.623
*Log2 (Relative protein abundance at hospital admission; RFU)*			
Interleukin-8	11.1 (0.5)	11.4 (0.8)	< 0.001
Advanced glycosylation end product-specific receptor, soluble	10.9 (1.2)	11.8 (1.1)	< 0.001
Interleukin-6	9.7 (0.8)	10.0 (0.9)	0.032
Jun dimerization protein 2	8.1 (0.3)	8.0 (0.2)	0.039
Retinol-binding protein 4	14.0 (0.4)	14.0 (0.5)	0.287
Adiponectin	11.3 (0.6)	11.4 (0.6)	0.343
Leptin	14.8 (1.2)	15.0 (1.1)	0.496
Angiotensinogen	13.7 (0.3)	13.7 (0.4)	0.648
Mitogen-activated protein kinase 9 (JNK2)	13.2 (0.5)	13.2 (0.4)	0.671
Mitogen-activated protein kinase 8 (JNK1)	9.6 (0.3)	9.6 (0.3)	0.911
Leptin/adiponectin	1.3 (0.1)	1.3 (0.1)	0.980

Severe hyperglycaemia was defined as grade 3 or higher hyperglycaemia according to the Common Terminology Criteria for Adverse Events (CTCAE) version 5.0. Chi-square tests were applied for categorical variables, Mann–Whitney *U* tests for non-normally distributed variables and unpaired *t*-tests for normally distributed variables

BMI, Body Mass Index; HOMA-IR, Homeostatic Model Assessment for Insulin Resistance; IQR, interquartile range; no., number; RFU, relative fluorescent units; SD, standard deviation

Based on our findings, we hypothesize that following SARS-CoV-2 infection, imatinib treatment mitigates the incidence of hyperglycaemia by enhancing peripheral glucose uptake. This effect could be mediated either through direct attenuation of disease severity or by improving biomarkers associated with peripheral insulin resistance (Fig. 1D). The latter could be exerted through attenuation of JNKs and inflammation. Moreover, imatinib treatment resulted in improved adiponectin levels, suggesting attenuation of adipose tissue inflammation and thus dysfunction (Fig. 1D). JNK serves as an important mediator of cellular stress responses and inflammation, and drives obesity-induced insulin resistance [7]. Previous studies have demonstrated that imatinib improves insulin signalling by reducing JNK activity in patients with diabetes mellitus [8]. Imatinib has also been shown to promote adipocyte differentiation and increase adiponectin levels in patients with cancer [9]. In addition, imatinib enhances microvascular perfusion, facilitating glucose transport to peripheral tissues [10]. While our study did not demonstrate a significant increase in ACE2 enzymatic activity, imatinib did lower Ang-II levels. Ang-II serves as a potent activator of JNK, and additionally exerts pro-inflammatory and vasoconstrictive effects [11]. Therefore, reducing Ang-II levels could enhance peripheral glucose uptake. In line with a previous study, COVID-19 patients in our study had preserved beta cell function, as indicated by high C-peptide levels [1]. This supports their findings that in COVID-19, hyperglycaemia results from peripheral insulin resistance rather than beta-cell failure. On the other hand, imatinib did not affect biomarkers associated with AMPK and PPAR-*α* signalling pathways, pancreatic function, gluconeogenesis or the renin–angiotensin–aldosterone system. As evidenced by the changes in IL-6, JNK and adiponectin, the effect of imatinib on glucose uptake appears to be predominantly related to the interplay between pro-inflammatory cytokines produced by polarized macrophages and adipokine secretion by adipocytes [5, 8]

In our study, imatinib did not affect glucose, insulin, C-peptide or HOMA-IR levels. While definitive conclusions are constrained because blood samples were typically collected under non-fasting conditions, our findings indicate that imatinib did not lower blood glucose levels across the entire study cohort of predominantly normoglycemic participants. Instead, imatinib specifically attenuated hyperglycaemic dysregulation. The apparent discrepancy with the reduced incidence of hyperglycaemia can be attributed to the fundamentally different mechanisms regulating glucose homeostasis in euglycemic conditions and those contributing to the development of insulin resistance and hyperglycaemia, particularly in the context of SARS-CoV-2-induced insulin resistance [12]. This distinction is exemplified by prior research demonstrating that JNK mediates obesity induced insulin resistance [8], whereas it exerts no influence on glucose metabolism in healthy lean mice [13]. In a previous study, we demonstrated that critical COVID-19 disease severity is associated with higher circulating IL-6 levels, along with a trend towards lower adiponectin levels [14]. However, because blood samples were most often not collected during the actual occurrence of hyperglycaemia (Fig. 1A), disturbances that occur with hyperglycaemic dysregulation in COVID-19 could not be established. Another notable limitation of our study is that aptamer assays capture relative protein abundance rather than absolute concentrations [15]. Consequently, we are unable to determine the absolute magnitude of the effect of imatinib.

In conclusion, our study demonstrates that hyperglycaemia is associated with increased disease severity, and that imatinib treatment reduces severe hyperglycaemia incidence in hospitalised patients with COVID-19. Imatinib did not affect markers of liver insulin resistance, beta-cell function and ACE-2 enzymatic activity. The observed attenuation of Ang-II, JNK signalling and inflammatory markers, coupled with enhanced adipokine secretion, suggests that the improved glycaemic regulation is linked to enhanced peripheral insulin sensitivity.

### Supplementary Information

**Additional file 1**. Supplementary table 1. Effect of imatinib on the abundance of glucoregulatory proteins.

## Data Availability

Pseudonymized patient data used for this study are available from the corresponding author (j.aman@amsterdamumc.nl) for other researchers when reuse conditions are met.

## Abbreviations

ACE2: Angiotensin-converting enzyme 2
Ang-II: Angiotensin-II
Ang(1-7): Angiotensin-(1-7)
CTCAE: Common Terminology Criteria for Adverse Events
COVID-19: Coronavirus disease 2019
ELISA: Enzyme-linked immunosorbent assays
HOMA-IR: Homeostatic Model Assessment for Insulin Resistance
HR: Hazard ratio
JNK: C-Jun N-terminal protein kinase
SARS-CoV-2: Severe acute respiratory syndrome coronavirus 2
